# *ZATELLITE:* a toolkit to visualize and manipulate human centromeres in live cells using synthetic zinc fingers

**DOI:** 10.64898/2026.08.04.742839

**Published:** 2026-08-05

**Authors:** Nestor Saiz, Aleah Goldberg, Finnegan Clark, Guohong Tong, Manjunatha Kogenaru, Peter H Whitney, Osama Abdin, Fabio Alfieri, Philip M. Kim, Glennis A. Logsdon, Liam J. Holt, Jef D. Boeke, Marcus B. Noyes, Teresa Davoli, Timothee Lionnet

## Abstract

Changes in the number of chromosomes or their spatial organization within the nucleus have critical consequences for cell fate. Yet capturing the karyotype or three-dimensional architecture of chromatin in living cells remains limited by the difficulty in labeling endogenous loci non-invasively. The most widely used tools rely on dCas9, a bulky protein whose persistent DNA binding interferes with DNA and RNA metabolism, causes DNA damage, and is hard to multiplex. We developed *ZATELLITE* , an AI-enabled tool to target endogenous repetitive sequences with fluorescently-tagged synthetic zinc fingers. *ZATELLITE* probes have key advantages over dCas9: they are smaller, easier to multiplex and, critically, they do not cause DNA damage or chromosomal abnormalities, even after long-term labeling. We generated a collection of *ZATELLITE* probes to label the centromeres of nearly all human chromosomes, enabling the capture of genome organization and karyotype alterations in real time in living cells. Finally, we show that *ZATELLITE* can be used for simultaneous labeling and epigenetic editing of centromeres. Thus, *ZATELLITE* is a non-toxic, versatile tool for genome visualization and manipulation.

## Full Text Availability

The license terms selected by the author(s) for this preprint version do not permit archiving in PMC. The full text is available from the preprint server.

